# Characterization of Neutral Lipids of the Oleaginous Alga *Micractinum inermum*

**DOI:** 10.3390/molecules29020359

**Published:** 2024-01-11

**Authors:** Arjun H. Banskota, Joseph P. M. Hui, Alysson Jones, Patrick J. McGinn

**Affiliations:** Aquatic and Crop Resource Development Research Centre, National Research Council Canada, 1411 Oxford Street, Halifax, NS B3H 3Z1, Canada; joseph.hui@nrc-cnrc.gc.ca (J.P.M.H.); alysson.jones@nrc-cnrc.gc.ca (A.J.); patrick.mcginn@nrc-cnrc.gc.ca (P.J.M.)

**Keywords:** biofuels, fatty acid, lipid, *Micractinum inermum*, microalgae, neutral lipid, triacylglycerols, UHPLC-HRMS

## Abstract

An oleaginous microalga *Micractinum inermum* isolated from Mariana Lake, AB, Canada was cultured in a 1000 L photobioreactor with an f/2 medium to study its lipid content and neutral lipid profile. Algal biomass was collected at the stationary phase contained a significant amount of lipids (44.2%), as determined by Folch’s method. The lipid was fractionated into neutral lipid, glycolipid and phospholipid fractions. The neutral lipid constitutes almost 77.3% of the total lipid species and is mainly composed of triacylglycerols (TAGs) determined by a proton NMR study. UHPLC-HRMS analysis allows us for the first time to identify 81 TAGs in the neutral lipid fraction of *M. inermum*. The fatty acid acyl side chains were identified based on fragment ions observed in MSMS analysis. TAGs with fatty acid acyl chains 18:1/18:1/18:1, 18:1/18:1/16:0, 18:2/18:1/16:0, and 18:2/18:2/18:0 were the major ones among the identified TAGs. Fatty acid analysis further supports the fact that oleic acid was the major fatty acid present in the neutral lipid fraction of *M. inermum* constituting 41.7%, followed by linoleic acid at 21.5%, and palmitic acid at 21.2%. The saturated and monounsaturated fatty acids were 67.8% or higher in the lipid fraction. Long-chain fatty acids were only present in a minor quantity. The results clearly demonstrate that *M. inermum* is an excellent source for TAGs.

## 1. Introduction

*Micractinum inermum* is a green microalga found in freshwater environments such as ponds, lakes, and streams. Microalgae are known for their diverse ecological roles, from being a source of food for various aquatic organisms to being valuable bioactive molecules for human beings [1]. Astaxanthin and β-carotene are two examples of carotenoids produced commercially from microalgae, *Haematococcus pluvialis* and *Dunaliella salina*, respectively [2,3]. These microalgal pigments are used in feed, food, and health applications [4]. Because of high lipid content, they are also considered as a potential source of next-generation biofuels [5]. Several studies have been conducted on the cultivation of *M. inermum* to maximize biomass and lipid productivity using a blended wastewater medium [6,7,8]. *M. inermum* produces a high content of C16–C18 fatty acids which are useful as biofuels with lipids composed of approximately 88% triacylglycerols (TAGs) [6]. Because of the lipophilicity and complex nature of neutral lipids, no study has been conducted on the characterization of those TAGs in *M. inermum.*

Triacylglycerol (TAG) is composed of three fatty acids and a glycerol backbone (also known as triglycerides). They are the primary form of stored energy in organisms and play crucial roles in biological systems [9]. They form part of cell membranes and are involved in maintaining membrane structure and fluidity [10]. These molecules are reported to have antioxidant properties, playing an important role in protecting cells from oxidative damage and even apoptosis [11]. Microalgal oils are considered a next-generation biofuel because they can be produced from photosynthetic organisms without using sugar, starch, or edible oil feedstocks. Transesterification of TAG-rich algal oil with methanol or ethanol is accomplished using a catalyst to produce biodiesel. The quality of algal biodiesel depends upon the characteristics of the fatty acids and includes parameters such as chain length and degree of unsaturation [12].

Microalgae can grow both in outdoor and enclosed environments, allowing manipulation of the culture conditions to enhance the production of target metabolites. Numerous studies have been conducted to enhance lipid production in green microalgae for possible biofuel production. Rattanapoltee and Kaewkannetra (2014) studied the lipid productivity of *Chlorella vulgaris* under autotrophic, heterotrophic, and mixotrophic conditions. Even though the highest amount of lipid production (32.9%) was observed under heterotrophic conditions, the most energy-efficient system in terms of lipid production was the mixotrophic [13]. Lipid production and its regulation in microalgae are species-specific and influenced by environmental conditions such as nutrients, salinity, and pH [14]. Under fed-batch modes, *Scenedesmus incrassulatus* was reported to produce a maximum biomass of 5.65 gL^−1^ with lipid contents of 58.27% when biodiesel-derived glycerol waste was used as the carbon source in mixotrophic conditions in a vertical tubular photobioreactor [15]. Based on a mathematical model of microalgal growth and a lipid accumulation study in six different cultivation conditions (i.e., batch (B), continuous (C), fed-batch, semi-batch, B and B two-stage, and C and B two-stage), the semi-batch operation (without stress) as well as the C and B two-stage operation (with stress) showed the best performance in terms of lipid productivity [16]. In the present investigation, we undertake a thorough examination of the neutral lipid composition of *M. inermum*, cultivated within a 1000 L photobioreactor (PBR). Our overall aim was to provide a detailed characterization of triacylglycerols (TAGs) in this strain using cutting-edge ultra-high-performance liquid chromatography coupled with high-resolution mass spectrometry (UHPLC-HRMS).

## 2. Results

### 2.1. M. inermum and Biomass Collection

**Figure 1:**
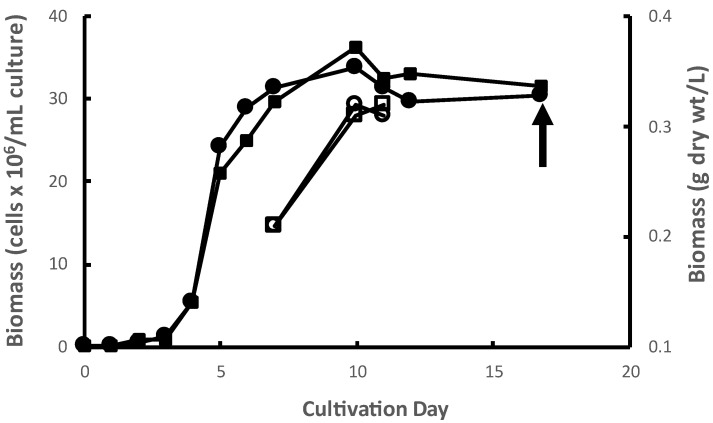
Growth curves of *M. inermum* in duplicate 1000 L PBRs. Accumulation of microalgae cells in PBR 1 (filled circles) and PBR 2 (filled squares). Corresponding accumulation of biomass dry weight in PBR 1 (open circles) and PBR 2 (open squares) on days 7, 10, and 11.

*M. inermum* was cultured in duplicate 1000 L photobioreactors (PBRs) in batch mode. The mean exponential growth rate (Figure 1) and yield from the duplicate PBRs was 1.55 d^−1^ and 0.36 g dry biomass L^−1^ of culture, respectively. The growth rates observed were typical of this species (Dickinson et al. 2019 observed growth rates ranging from 1.4 to 1.6 d^−1^ under conditions comparable to those used in this study) [17]. The observed yields of algal biomass in a PBR are highly dependent on many factors including nutrient regime, effective light intensity, temperature, pH, and gas mixing dynamics, among others. Biomass yields under autotrophic conditions, i.e., photosynthetic as conducted here, tend to be lower, sometimes much lower, than other cultivation modes like mixotrophy and heterotrophy under comparable conditions of pH, temperature, etc. Nitrogen, phosphorus, and carbon concentrations, in particular, exert strong control over yields under autotrophic conditions like those used here. The use in different studies of different growth media, and therefore different nutrient concentrations, in different PBR systems confound comparisons of yields across studies. However, the yields observed in the present study are fairly typical of autotrophic algal cultures grown in fairly lean media like f/2 [18,19]. The microalgal biomass was collected at the stationary phase by centrifugation and then freeze-dried. Freeze-dried algal biomass of *M. inermum* is light green in color as shown in Figure S1.

### 2.2. Moisture and Total Lipid Content

The total lipid and moisture content of the freeze-dried biomass were measured and the results are shown in Table 1. The moisture content of the freeze-dried biomass was 5.8% and the lipid content was 44.2%, as measured by Folch’s method [20]. The lipid was dark black in color possibly due to the presence of pigments, especially chlorophylls [17]. This was confirmed by HPLC analysis, which showed multiple signals belonging to chlorophylls and possibly degradation products determined by their UV spectrum (Figure S2). The proton NMR spectrum of the total lipid showed signals assigned to unsaturated protons, sugars, glycerol moieties, and fatty acids (Figure S3).

### 2.3. Separation of Lipid Classes and ^1^H NMR Analysis

**Figure 2:**
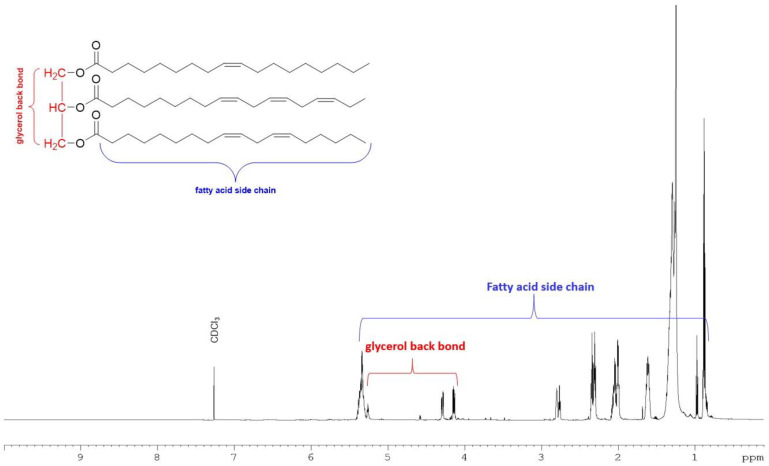
^1^H NMR spectrum of neutral lipid fraction eluted with chloroform in SPE recorded in 700 MHz NMR with CDCl_3_ with a representative structure of TAG.

The total lipid extracted from *M. inermum* was further partitioned into three fractions based on the polarity using silica gel-based solid phase extraction (SPE). The SPE fractions successively eluted with chloroform, acetone, and methanol are primarily composed of neutral lipids, glycolipids, and phospholipids, respectively, based on the method described by Ryckebosch et al., 2011 [21]. The neutral lipid accounted for 77.3% followed by glycolipids (13.2%) and phospholipids (5.6%) (Table 1). The major signals observed in the proton NMR spectrum of the neutral lipid fraction (Figure 2) belonged to triacylglycerols (TAGs) [22]. The NMR spectrum of glycolipid and phospholipid fractions showed signals mainly for sugars and some fatty acids indicating the potential presence of polar lipids in those fractions (Figures S4 and S5).

### 2.4. Triacylglycerols (TAGs) Analysis of Neutral Lipid Fraction

**Figure 3:**
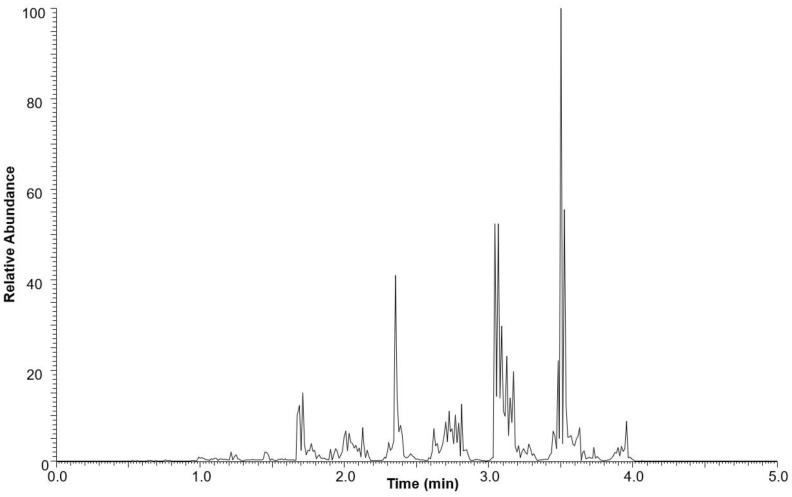
Base peak chromatogram of the neutral lipid fraction of *M. inermum* in positive mode ESI-MS.

The neutral lipid fraction was further analyzed for triacylglycerol content using ultra-high-performance liquid chromatography high-resolution mass spectrometry (UHPLC-HRMS). The total ion current (TIC) chromatogram of the neutral lipid fraction is shown in Figure 3. TAGs were eluted between 1.0 and 5.0 min.

In total, 81 TAGs were detected in the neutral lipid fraction of *M. inermum* and are listed in Table 2, along with accurate mass measurement and their retention time. TAGs were predominantly ionized as ammonium adducts [M + NH_4_]^+^ [23], their relative intensities of individual molecular ion peaks were measured and a heat map analysis was performed. Based on the heat map results, as shown in Table 2, TAGs with fatty acyl side chains 18:1/18:1/18:1, 18:1/18:1/16:0, 18:2/18:1/16:0 and 18:2/18:2/18:0 were the major ones among the identified TAGs. On the other hand, only a few TAGs with a fatty acid side chain containing 14:0 and 14:1 were detected, while one TAG with a long chain fatty acid side chain was identified. A series of unknown TAGs detected with possible molecular formulas are also listed in Table 2.

### 2.5. Fatty Acid Analysis of Lipid Fractions

**Figure 4:**
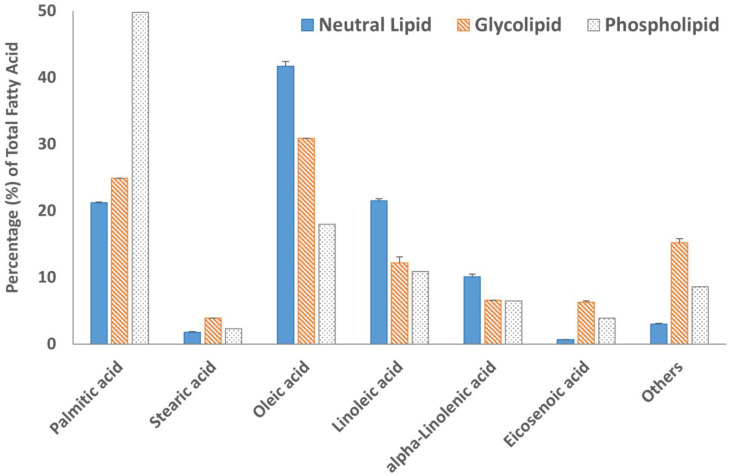
The fatty acid profile of neutral lipid, glycolipid, and phospholipid fractions and results are expressed in the percentage of individual fatty acids in the total fatty acids observed. Data are from duplicate experiments, except for the phospholipid fraction, which was carried out in a single experiment.

**Figure 5:**
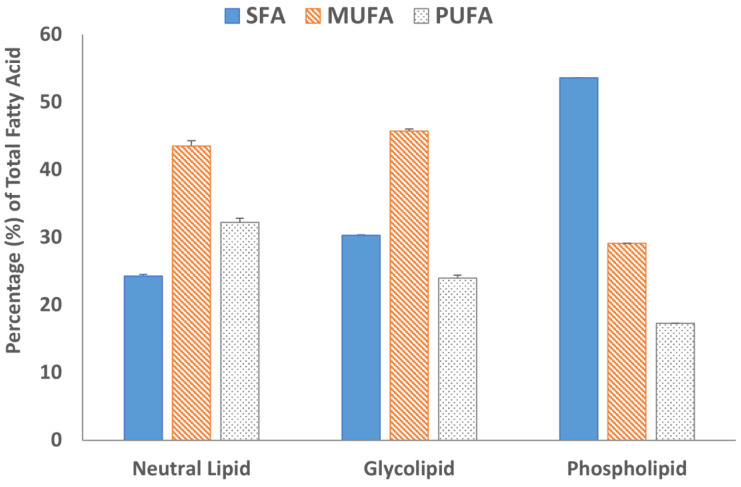
Percentage distribution of saturated fatty acid (SFA), monosaturated fatty acids (MUFA), and polyunsaturated fatty acid (PUFA) in the lipid fractions. Data are from duplicate experiments, except for the phospholipid fraction, which was carried out in a single experiment.

Fatty acid methyl esters (FAMEs) of all three lipid fractions were analyzed by gas chromatography after transesterification using a BF_3_ solution. The results are summarized in Figure 4 and Figure 5. The GC chromatograms are shown in Figure S6. Oleic acid was the major fatty acid present in the neutral lipid and glycolipid fractions containing 41.7% and 30.9% of the total fatty acids within the fraction, respectively (Figure 4). Palmitic acid was the major fatty acid accounting for almost 50% of the total fatty acid present in the phospholipid fraction. The second most abundant fatty acid in the neutral lipid fraction was linoleic acid (21.5%), followed by palmitic acid (21.2%). Similarly, the glycolipid fraction contained 24.9% palmitic acid and only 12.2% linoleic acid. Overall, saturated (SFA) and monounsaturated fatty acid (MUFA) concentrations were 67.8% or higher in all three lipid fractions (Figure 5). The absolute values of individual fatty acids (mg/g of the lipid fraction) are listed in Table S1.

## 3. Discussion

Microalgae are a diverse group of organisms capable of performing photosynthesis. The photosynthetic capability of these microorganisms gives them extraordinary potential as possible biological tools to capture industrial CO_2_. They are also considered as energy crops for biofuels because of their high lipid content [5,24,25]. These microorganisms are also equally capable of producing a wide range of commercially interesting products such as fats, sugars, carotenoids, and many others [26]. *M. inermum* is a freshwater microalga well studied for the production of lipids especially for biodiesel [6,7,8]. Shi et al. (2019) reported that *M. inermum* JL 1, a locally collected strain, was capable of growing robustly under photoautotrophic, mixotrophic, and heterotrophic culture conditions. Lipid accumulation of the *M. inermum* JL 1 strain was around 52% of dry biomass and achieved a high lipid productivity of 0.68 g L^−1^ d^−1^ [6]. In our study we observed 44.2% lipid content in the M. *inermum* freeze-dried biomass (Table 1), somewhat lower but still significantly higher when compared to another possible biofuel feedstock such as agriculture waste. Park et al. (2017) studied the *M. inermum* NLP-F014 strain for cost-effective biomass production using a blended wastewater medium, which produces 0.95 g-dry cell weight (DCW) L^−1^d^−1^ [7]. The maximum biomass production of the *M. inermum* NLP-F014 strain 1.03 g-DCW L^−1^d^−1^ was achieved when influent collected from a domestic wastewater treatment plant and the liquid fertilizer from a swine wastewater treatment blend with Bold’s Basal Medium with a ratio of 5–15% (*v*/*v*) [8]. These findings clearly demonstrated the potential of *M. inermum* as a next energy crop using wastewater as a nutrient source. Another study of *M. inermum* reported that growth rates were maximized by supplementation of the air stream with 1% CO_2_, relative to 5% or 10% CO_2_, under blue, red, blue/red mixtures, and white lights (Dickinson et al. 2019) [17].

In the current study, we have used an f/2 medium to culture *M. inermum* in a 1000 L photobioreactor and harvested algal biomass in the stationary phase by centrifugation. To understand the composition of the lipid, the total lipid extracted from the freeze-dried algal biomass was further fractionated into three classes based on their polarities using SPE as described previously by Ryckebosch et al. 2011 [21]. The neutral lipid fraction eluted with chloroform constitutes 77.3% of the total lipid followed by the glycolipid fraction (13.2%) and the methanol fraction (5.6%) (Table 1). Signals in the proton NMR spectrum (Figure 2) of the neutral lipid fraction clearly demonstrated that the fraction is composed of mainly triacylglycerols (TAGs) [22]. The oxygenated methine signal at 5.25 ppm and two oxygenated methylene signals between 4.10 and 4.40 ppm belong to glycerol moiety [22]. The presence of multiple double-bond protons at 5.27 ppm and multiple methylene signals at 2.80 and 2.30 ppm strongly suggested that the fatty acid acyl chain contains one or more double bonds. The results are inconsistent with the previous report by Shi et al. (2019) that the TAGs content would be around 88% of the total lipids in *M. inermum* when the culture medium was supplemented with salt [6]. Usually, microalgae accumulate TAGs under stress conditions. Li et al. (2012) studied gene expression on *M. pusillum* under nitrogen (N) starvation which accumulate significant amounts of TAGs and observed 290 expressed sequence tags (ETSs) that showed significant differential expression compared to a control group [27]. Abu-Ghosh et al. (2019), on the other hand, found that sethoxydim-resistant mutants of *Micractinium* sp. displayed a significant increase in TAG cell content to 84% without compromising the growth rate even in non-stressful conditions [28]. Even though *M. inermum* has been reported to produce mainly TAGs, no study has been conducted for the characterization of intact TAGs, probably because of its lipophilicity and complex nature. Identification of the intact TAGs in *M. inermum* may be valuable for researchers to make strategies for biodiesel production or for use in other consumer products, especially in the cosmeceutical industry as a source of bioactive lipids. The proton NMR spectrum of acetone and MeOH fractions showed either trace or no TAG signals (Figures S4 and S5).

The TAG composition of the neutral lipid fraction was studied by UHPLC-HRMS. The separation of TAGs was achieved using Thermo Hypersil Gold C8 column (100 × 2.1 mm, 1.9 μm) as described previously by Banskota et al. (2022) [29]. Electrospray ionization (ESI) was used to characterize TAGs present in the neutral lipid. ESI is a soft ionization technique widely used to characterize various classes of lipids present both in microalgae and seed oils [23,30,31,32]. The LC separation was performed over ten minutes with all TAGs eluted within the first five minutes, as shown in the total ion current (TIC) chromatogram (Figure 3). ESI generates intact molecular ions, typically observed as ammonium, sodium, or proton adducts. The ammonium adduct ions were selected for identification of TAGs using the LipidMAPS database [33] because they produced prominent fragment ions leading to the structure elucidation of TAGs. A list of TAGs with observed [M + NH_4_]^+^ ions matched with the database is presented in Table 2. Altogether 33 ions were verified as TAGs.

**Figure 6:**
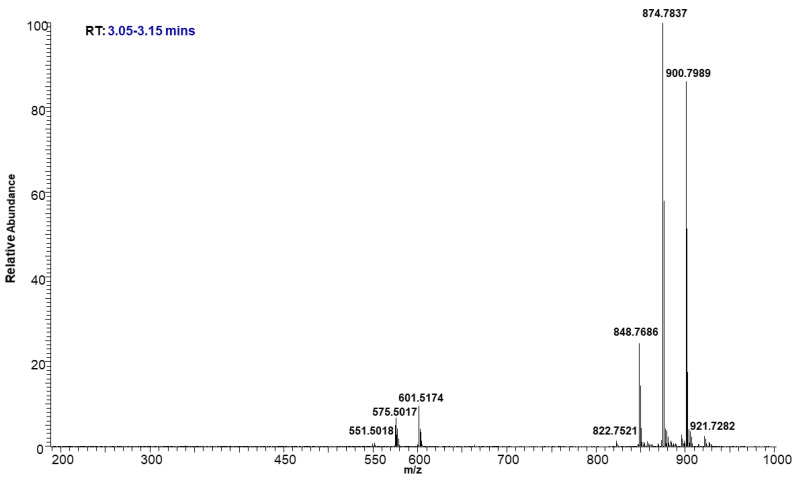
Representative mass spectrum of TAGs eluted at 3.05–3.15 min.

The molecular ion of TAG mostly fragmented under low energy collision-induced dissociation (CID) in a systematic fashion into diacylglycerol (DAG) product ions. The observed DAG product ions, through neutral loss of fatty acid, allowed for the confident identification of TAGs and the assignment of the fatty acid acyl side chain. Even though some of these TAGs were not chromatographically resolved, their identifications were incontrovertible based on the accurate mass. For example, Figure 6 shows the precursor ions at *m/z* 848.7686, 874.7837, and 900.7989, eluting between 3.05 to 3.15 min as shown in the TIC (Figure 3). The intensity of each molecular ion was measured. Based on the intensities of the individual ammonium adduct ions we were able to create a heat map of the TAGs within the neutral lipid fractions to compare their relative abundance. The relative percentages of individual ammonium adduct ions are listed in Table 2.

**Figure 7:**
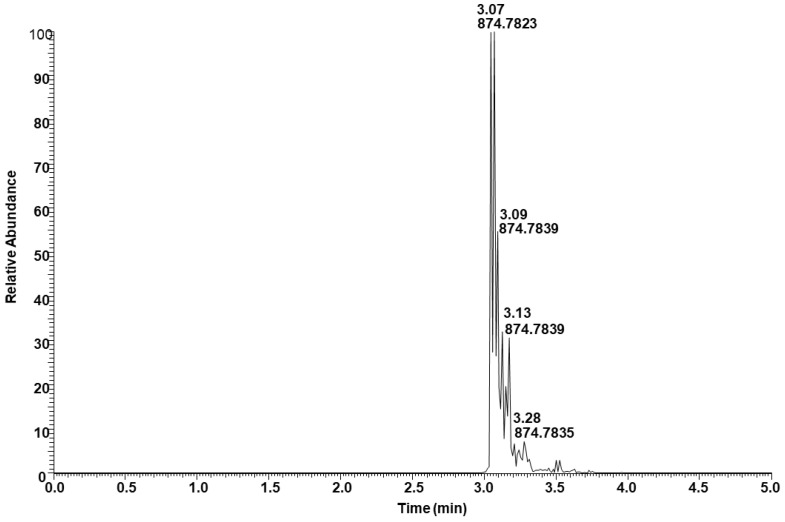
Total ion extracted for TAG with [M + NH_4_]^+^ ion at *m*/*z* 874.7837.

**Figure 8:**
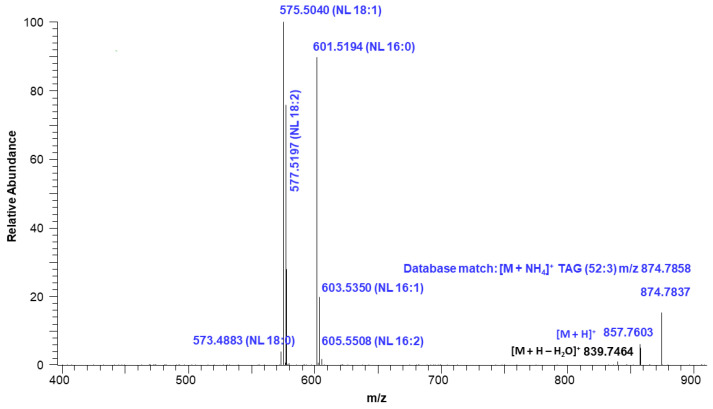
Fragmentation ions corresponding to neutral loss (NL) of TAG (52:3) with a molecular ion at *m*/*z* 874.7837.

Figure 7 shows the extracted ion intensity of a TAG with [M + NH_4_]^+^ ion at *m*/*z* 874.7837, which was matched with the TAG database (C52:3) with *m*/*z* 874.7858 [33]. The major fragments observed in the MSMS spectra represent the neutral losses of fatty acids from the glyceride backbone. For example, the precursor ion at *m*/*z* 874.7837 first lost an ammonium adduct and further gave major fragments at *m*/*z* 601.5194, 577.5197, and 575.5040, representing the neutral loss of fatty acids16:0, 18:2, and 18:1, respectively (Figure 8). These fragment ions lead to the conclusion that the structure of the TAG is 18:2/18:1/16:0. Minor ion peaks at *m*/*z* 605.5508, 603.5350, and 573.4883 due to neutral loss of 16:2, 16:1, and 18:0 fatty acids further suggested the presence of TAGs 18:2/18:0/16:1 and 18:0/18:1/16:2 in lower abundance having identical molecular ion and retention time as the major TAG 18:2/18:1/16:0. The structure of a TAG with a molecular ion at *m*/*z* 900.7989 was identified as a mixture of 18:2/18:2/18:0 (major) and 18:3/18:1/18:0 (minor) based on the fragment ion observed (Figure S7). Similarly, a TAG with molecular ion at *m*/*z* 848.7686 was identified as a mixture of 18:2/16:0/16:0 and 18:1/16:1/16:0 (Figure S8). Determination of the position of individual fatty acyl chains within the glyceride backbone was outside the scope of this study. A series of minor unknown TAGs with molecular ion [M + NH_4_]^+^ peaks at *m*/*z* 930.7733, 932.7922, and 934.8069 were also detected and matched with the database A molecular mass suggested that one or more fatty acid side chains may have total two hydroxy groups. An MSMS spectrum of one of the TAGs with a molecular ion at *m*/*z* 930.7733 is shown in Figure S9.

The heat map diagram of individual TAGs (Table 2) strongly suggested that TAGs with fatty acyl chains 18:1/18:1/18:1, 18:1/18:1/16:0, 18:2/18:1/16:0, and 18:2/18:2/18:0 were most abundant in *M. inermum* constituting more than 40% of the total TAGs. Fatty acids 18:1, 18:2, and 16:0 were identified as oleic acid (18:1 n-9), linoleic acid (18:2 n-6), and palmitic acid (16:0), respectively, based on the fatty acid analysis. The fatty acid analysis revealed that oleic acid was the major fatty acid found in the neutral lipid fraction of *M. inermum* constituting 41.7%, followed by linoleic acid (21.5%), and palmitic acid (21.2%). This further confirmed that the TAGs having these fatty acyl chains (C18:2, C18:1, and C16:0) are the major TAGs in *M. inermum*. Only a handful of TAGs containing 14:0 and 14:1 were detected. Even though longer chain fatty acids, i.e., C:20, C:22, C:24, and C:24 were detected in minor quantities, only one TAG with those fatty acids was detected. It is worth mentioning that *M. inermum* cultured in an f/2 medium was shown to produce the highest percentage of monounsaturated fatty acids (MUFAs), which are desirable for biodiesel, followed by saturated fatty acids (SFAs) which are also shown to be of great importance for biodiesel production [12]. In a previous study, Shi et al. (2019) observed higher PUFA (55.7%) content which suggested the importance of the medium selection and the culture condition in influencing the TAGs and the fatty acid composition [6].

## 4. Materials and Methods

### 4.1. General

The ^1^H NMR spectra were measured on a Bruker (Billerica, MA, USA) 700 MHz spectrometer using a 5 mm cryogenically cooled probe. High-resolution mass spectra were acquired using a Thermo Fisher Scientific (Waltham, MA, USA) Q Exactive^TM^ Hybrid Quadrupole-Orbitrap^TM^ Mass Spectrometer. Gas chromatography (GC) analyses were carried out on an Agilent (Santa Clara, CA, USA) Technologies 7890A GC spectrometer with an FID detector using an Omegawax 250 fused silica capillary column (30 m × 0.25 mm × 0.25 μm film thickness). Supelco^®^ 37 component FAME mix and PUFA−3 (Supelco, Bellefonte, PA, USA) were used as fatty acid methyl ester standards. HPLC-grade solvents were used for the extraction and analyses.

### 4.2. M. inermum Culture

*M. inermum* AB07-I-F-PLM was isolated from Mariana Lake, Alberta, Canada (55.9542° N, 112.0181° W). In brief, raw water samples were shipped back to the lab on ice. Once received, aliquots of the field samples were transferred into individual wells of multi-well plates and amended with fresh, sterile f/2 growth media (Guillard 1975) modified by the addition of Na_2_SeO_3_ 5H_2_O (sodium selenite) to a final concentration of 1 × 10^−11^ M [34]. Multi-well plates were placed on an orbital shaking platform in an illuminated incubator and gently shaken under continuous aeration with humidified air enriched to 5% CO_2_ (*v*/*v*). Plates were incubated for up to 14 days without further intervention. After this period, samples from wells with obvious algal growth, indicated by the appearance of pigments, were transferred to solidified growth media and streaked to isolate individual colonies. The isolate was identified at species level by performing 18S and associated ITS sequence analysis of the ribosomal DNA gene obtained from unialgal axenic cultures as described by Park et al. (2012) [35]. For scale-up into 1000 L PBRs, colony material was picked with a sterile toothpick and transferred into sterilized liquid media in flasks and grown for a period of 10–14 days, followed by transfer into 20 L carboys filled with the same growth medium for a further 10–14 days. The carboy culture was used to inoculate duplicate 1000 L Brite-Box™ photobioreactors as described previously by Banskota et al. 2012 [36]. In brief, the cultivation medium was composed of lake water (0.35 μm filtered, UV treated, and pasteurized for a minimum of 8 h at 85 °C from Kearney Lake, Bedford, Nova Scotia) amended with f/2 nutrients on the first day of cultivation. Culture pH was maintained at 7.5 by the periodic addition of carbon dioxide. Twenty-four hours of continuous illumination was provided by 40, 32 W full spectrum T8 (6500 k) fluorescent lamps (Alto Technology, Philips Lighting, Mississauga, ON, Canada) distributed evenly throughout the volume of the culture. The temperature was maintained at 21 °C by flowing chilled water through a titanium cooling loop. After 17 days of batch cultivation, approximately 2000 L of the stationary phase culture was pooled and harvested with a process centrifuge (CEPA Z101H, Carl Padberg Zentrifugenbau GmbH, Lahr/Schwarzwald, Germany). The collected material was lyophilized for 36 h at a low shelf temperature (<5 °C) in a large-capacity freeze-dryer (model 35EL, VirTis, Gardiner, NY, USA) resulting in a total yield of 720 dry g of biomass. The freeze-dried biomass was stored at room temperature in an airtight plastic container until lipid extraction.

### 4.3. Total Lipid Content

Total lipid content was determined by the Folch method with slight modification [10]. In brief, the freeze-dried algal biomass was ground manually and filtered through a laboratory sifter (Buhler AG, Uzwil, Switzerland) to collect flour with particle size < 0.5 mm for further analysis. The sample (~100 mg) was extracted at room temperature, then homogenized with chloroform/methanol (2:1, 1 mL × 3) using a bead beater (Bead Mill_24_, Fisher Scientific, Hampton, NH, USA) in a 2 mL Lysing matrix Y tubes (3 × 1 min cycles) in triplicate. The combined lipid extracts were dried under nitrogen and kept under vacuum overnight, weight was measured gravimetrically and total lipid content was calculated using the following formula.

Total lipid (%) = weight of lipid/weight of sample × 100(1)

### 4.4. Lipid Class Separation by Solid Phase Extraction (SPE)

The total lipid was fractionated into three different classes of lipid, i.e., neutral lipids containing mostly triglycerides (TAGs), glycolipids, and phospholipids following the previously reported solid phase extraction (SPE) method by Ryckebosch et al. 2011 [21]. In brief, the SPE column (Discovery DSC-Si Tube 3 mL 500 mg) was conditioned with 10 mL of chloroform. Approximately 100 mg of oil in 1.0 mL of chloroform was applied to the column. The column was then eluted successfully with chloroform (10 mL), acetone (10 mL), and methanol (10 mL), yielding neutral lipids, glycolipids, and phospholipids, respectively. The percentages of each class of lipid were determined by their weight taken gravimetrically after drying under nitrogen followed by further drying under vacuum overnight.

### 4.5. Triacylglycerols (TAGs) Analysis

UPLC-HRMS data were acquired on an UltiMate 3000 system coupled to a Q-Exactive^TM^ hybrid Quadrupole Orbitrap Mass Spectrometer (Thermo Fisher Scientific, Waltham, MA, USA) equipped with a HESI-II probe for electrospray ionization (ESI) as described previously by Banskota et al. 2022 [29]. Thermo Hypersil Gold C8 column (100 × 2.1 mm, 1.9 μm) was used for TAG separation at 40 °C with an acetonitrile/isopropyl alcohol (IPA) gradient. The initial gradient was 100% acetonitrile, which increased linearly to 5% IPA in 1 min, and then linearly to 70% IPA in 8 min, held for 2 min, at a flow rate of 750 µL/min. Through a flow splitter, approximately 1/15 of LC eluent was sent to the mass spectrometer. A makeup solution consisting of 5mM ammonium formate in IPA/deionized/methanol 1/2/7 (*v*/*v*) was delivered constantly at 100 µL/min to MS. MS data were acquired in positive ion mode which alternates between full MS and DataDependent MSMS scans, where the three most abundant precursor ions were subjected to MSMS using a 25 eV collision energy. The source parameters were set as follows: sheath gas, 15; auxiliary gas flow, 4; sweep gas, 0; spray voltage, 2.1 kV; capillary temperature, 375 °C; heater temperature, 300 °C.

### 4.6. Fatty Acid Analysis

Fatty acid analysis was conducted according to the Association of Official Analytical Collaboration (AOAC) official method 991.39 (AOAC, 2000) with slight modifications [37]. In brief, ~10–30 mg of lipid fractions were placed in a dry 5 mL screw-capped reaction vial and MeOH (1.0 mL) containing 0.1 mg of methyl tricosanoate as an internal standard (IS). The mixture was sonicated and 1.5N NaOH solution in MeOH (0.5 mL) was added, blanketed with nitrogen, heated for 5 min at 100 °C, and cooled for 5 min. A BF_3_ 14% solution in MeOH (1.0 mL, Sigma-Aldrich, St. Louis, MO, USA) was added, mixed, blanketed with nitrogen, and heated at 100 °C for 30 min. After cooling, the reaction was quenched by the addition of water (0.5 mL) and the FAME extracted with hexane (2.0 mL). Part of the hexane layer (300–600 μL) was transferred to a GC vial for analysis by GC-FID. GC-FID was carried out on an Agilent Technologies 7890A GC spectrometer using an Omegawax 250 fused silica capillary column (30 m × 0.25 mm × 0.25 μm film thicknesses) for fatty acid analysis. Supelco^®^ 37 component FAME mix and PUFA−3 (Supelco, Bellefonte, PA, USA) were used as fatty acid methyl ester standards. The fatty acid content in the lipid fractions was calculated by the following equation and expressed as mg/g sample. The experiment was performed in duplicate except for the phospholipid fraction which was carried out in a single experiment.

Fatty acid (mg/g) = (A_X_ × W_IS_ × CF_x_/A_IS_ × W_S_ × 1.04) × 1000 (2)

where, in Equation (2), A_X_ = area counts of fatty acid methyl ester; A_IS_ = area counts of internal standard (tricosylic acid methyl ester); CF_X_ = theoretical detector correlation factor is 1; W_IS_ = weight of IS added to the sample in mg; W_S_ = sample mass in mg; and 1.04 is the factor necessary to express the result as mg fatty acid/g sample.

## 5. Conclusions

In conclusion, we have studied the total lipid production of *M. inermum* isolated from Mariana Lake, AB, Canada in a 1000 L photobioreactor, which produces a significant amount of lipids at 44.4% of freeze-dried biomass mainly composed of TAGs. The neutral fraction containing TAGs was further characterized by UPLC-HRMS identifying 81 TAGs for the first time in *M. inermum*. TAGs with fatty acid acyl side chains 18:1/18:1/18:1, 18:1/18:1/16:0, 18:2/18:1/16:0, and 18:2/18:2/18:0 were the most abundant ones among the identified TAGs. The fatty acid analysis further indicated that oleic acid (41.7%) was the main fatty acid present in the neutral lipid fraction of *M. inermum* followed by linoleic acid (21.5%), and palmitic acid (21.2% of total fatty acids). The results suggest that *M. inermum* would be an excellent source for TAGs.

## Figures and Tables

**Table 1:** Moisture and lipid content of the *M. inermum* freeze-dried biomass. Results are expressed in percentages and the percentage of three different lipid classes are within the total lipid.

	Exp 1	Exp 2	Exp 3	Average ± SD
Moisture Content	5.6	6.0	5.9	5.8 ± 0.2
Lipid Content	44.0	44.5	44.0	44.2 ± 0.3
Neutral Lipid Fraction	79.0	74.4	78.6	77.3 ± 1.9
Glycolipid Fraction	14.3	14.2	11.0	13.2 ± 1.9
Phospholipid Fraction	4.0	6.2	6.6	5.6 ± 1.4

**Table 2:** Heat map of triacylglycerols (TAGs) identified in the neutral lipid fraction of *M. inermum.*

TAG Relative Abundance	RT (min)	Measured (*m*/*z*)	Matched or Exact ms (*m*/*z*)	Error (ppm) *	Formula	TAG C:DB	TAG Identity
0.41	1.45	838.6922	838.6919	−0.30	C_53_H_88_O_6_	50:7	18:3/16:3/16:1; 18:3/18:2/16:2; 18:2/16:3/16:2
0.60	1.45	864.7083	864.7076	−0.80	C_55_H_90_O_6_	52:8	18:3/18:1/16:4; 18:3/18:2/16:3; 18
0.77	1.67	930.7733	930.7756	2.48	C_57_H_100_O_8_	54:5; O2	NI
2.21	1.67	840.7084	840.7076	−0.95	C_53_H_90_O_6_	50:6	18:3/16:3/16:0; 18:3/16:2/16:1; 18:2/16:3/16:1; 18:2/16:4/16:0
3.28	1.71	866.7239	866.7232	−0.81	C_55_H_92_O_6_	52:7	18:3/18:1/16:3; 18:2/18:1/16:4
0.69	1.94	894.7557	894.7545	−1.34	C5_7_H_96_O_6_	54:7	18:3/18:2/18:3; 18:3/18:3/18:1
1.06	1.90	932.7922	932.7913	−0.96	C_57_H_102_O_8_	54:4; O2	NI
0.32	2.10	816.7093	816.7076	−2.08	C_51_H_90_O_6_	48:4	16:4/16:0/16:0; 16:3:16:1/16:0; 16:2/16:2/16:0
2.00	2.05	842.7249	842.7232	−2.02	C_53_H_92_O_6_	50:5	18:3/16:2/16:0; 18:3/16:1/16:1; 18:2/16:3/16:0; 18:2/16:2/16:1; 18:1/16:3/16:2; 18:1/16:4/16:0
3.09	2.06	868.7404	868.7389	−1.73	C_55_H_94_O_6_	52:6	18:3/18:2/16:1; 18:3/18:3/16:0; 18:1/18:0/16:5; 18:3/18:1/16:2; 18:1/18:1/16:4; 18:2/18:1/16:3
1.35	2.12	934.8069	934.8069	0.00	C_57_H_104_O_8_	54:3;O2	NI
0.68	2.35	818.7235	818.7232	−0.37	C_51_H_92_O_6_	48:3	16:3/16:0/16:0; 16:2/16:1/16:0
3.26	2.35	844.7389	844.7389	0.00	C_53_H_94_O_6_	50:4	18:1/16:3/16:0; 18:1/16:2/16:1; 18:2/16:2/16:0; 18:2/16:1/16:1; 18:3/16:1/16:0
5.08	2.35	870.7545	870.7545	0.00	C_55_H_96_O_6_	52:5	18:3/18:2/16:0; 18:2/18:2/16:1; 18:3/18:1/16:1; 18:2/18:1/16:2; 18:1/18:1/16:3; 18:0/16:2/16:3
3.40	2.35	896.7697	896.7702	0.56	C_57_H_98_O_6_	54:6	18:3/18:2/18:1
0.23	2.62	820.7403	820.7389	−1.71	C_51_H_94_O_6_	48:2	16:2/16:0/16:0 (major); 16:1/16:1/16:0 (minor); 18:2/16:0/14:0; 18:1/16:0/14:1; 18:1/16:1/14:0
4.70	2.75	898.7864	898.7858	−0.67	C_57_H_100_O_6_	54:5	18:3/18:1/18:1; 18:3/18:2/18:0; 18:2/18:2/18:1
1.75	2.78	846.7557	846.7545	−1.42	C_53_H_96_O_6_	50:3	18:3/16:0/16:0; 18:2/16:1/16:0; 18:1/16:1/16:1; 18:0/16:2/16:1
5.48	2.81	872.7709	872.7702	−0.80	C_55_H_98_O_6_	52:4	18:3/18:1/16:0; 18:2/18:2/16:0; 18:3/18:0/16:1; 18:3/18:0/16:1; 18:2/18:0/16:2; 18:1/18:0/16:3
11.46	3.07	900.8002	900.8015	1.44	C_57_H_102_O_6_	54:4	18:2/18:2/18:0 (major); 18:3/18:1/18:0 (minor)
13.01	3.07	874.7837	874.7858	2.40	C_55_H_100_O_6_	52:3	18:2/18:1/16:0 (major); 18:2/18:0/16:1 (minor); 18:1/18:0/16:2 (minor)
0.06	3.28	836.7695	836.7702	0.84	C_52_H_98_O_6_	49:1	18:1/16:0/15:0
3.50	3.07	848.7690	848.7702	1.41	C_53_H_98_O_6_	50:2	18:2/16:0/16:0; 18:1/16:1/16:0
0.43	3.28	862.7849	862.7858	1.04	C_54_H_100_O_6_	51:2	18:1/17:1:16:0; 18:2/17:0:16:0;
0.63	3.28	888.8000	888.8015	1.69	C_56_H_102_O_6_	53:3	18:2:/18:1/17:0
3.22	3.50	850.7862	850.7858	−0.47	C_53_H_100_O_6_	50:1	18:1/16:0/16:0
0.25	3.66	864.8025	864.8015	−1.16	C_54_H_102_O_6_	51:1	18:1/17:0/16:0
12.77	3.50	876.8016	876.8015	−0.11	C_55_H_102_O_6_	52:2	18:1/18:1/16:0
0.59	3.73	890.8178	890.8171	−0.79	C_56_H_104_O_6_	53:2	18:1/18:1/17:0
11.03	3.50	902.8167	902.8171	0.44	C_57_H_104_O_6_	54:3	18:1/18:1/18:1 (major); 18:2/18:1/18:0 (minor)
0.97	3.90	878.8174	878.8171	−0.34	C_55_H_104_O_6_	52:1	18:1/18:0/16:0
0.16	3.96	930.8485	930.8484	−0.11	C_59_H_108_O_6_	56:3	20:1/18:1/18:1 **
1.47	3.96	904.8329	904.8328	−0.11	C_57_H_106_O_6_	54:2	18:1/18:1/18:0 (major); 18:2/18:0/18:0 (minor)

NI—not identified, * Error ppm = (exact mass − measured mass)/exact mass × 10^6^, ** only neutral loss of FA 20:1 was observed, Low abundance 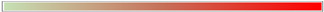 High abundance.

## Data Availability

Data are contained within the article and Supplementary Materials.

## Supplementary Materials

The following supporting information can be downloaded at: https://www.mdpi.com/article/10.3390/molecules29020359/s1, Figure S1: Freeze-dried algal biomass of *M. inermum* and total lipid extract; Figure S2: HPLC chromatogram of *M. inermum* lipid and UV spectrum of major peak; Figure S3: ^1^H NMR spectrum of total lipid extracted with chloroform/methanol (2:1) recorded in 700 MHz NMR Spectrometer; Figure S4: ^1^H NMR spectrum of glycolipid fraction eluted with acetone in SPE recorded in 700 MHz NMR Spectrometer; Figure S5: ^1^H NMR spectrum of phospholipid fraction eluted with methanol in SPE recorded in 700 MHz NMR Spectrometer; Figure S6: GC chromatogram of neutral lipid, glycolipid and phospholipid fractions after transesterification; Figure S7: Total ion extracted for TAG with [M + NH_4_]^+^ ion at *m*/*z* 848.7686 and fragmentation ions corresponding to neutral loss (NL) of TAG (50:2) with their fragmentation ions; Figure S8: Total ion extracted for TAG with [M + NH_4_]^+^ ion at *m*/*z* 900.7989 and fragmentation ions corresponding to neutral loss (NL) of TAG (54:4) with their fragmentation ions; Figure S9: Total ion extracted for unknown TAG with [M + NH_4_]^+^ ion at *m*/*z* 930.7733 and fragmentation ions corresponding to neutral loss (NL) of TAG (54:5; O2) with their fragmentation ions; Table S1: Fatty acid profile of lipid fractions. Results are expressed in mg/g biomass.
