# Supplementary material for: Characterization of Neutral Lipids of the Oleaginous Alga Micractinum inermum
Source: Molecules. 2024 Jan 11;29(2):359. doi: 10.3390/molecules29020359 (PMC10819927; doi:10.3390/molecules29020359)
Supplement: Supplementary file 1 [file molecules-29-00359-s001.zip › molecules-2791919-supplementary.pdf]

SUPPLEMENATRY DOCUMENTS

Characterization of Neutral Lipids of the Oleaginous Alga Micractinum inermum

*Arjun H. Banskota *, Joseph P. M. Hui, Alysson Jones and Patrick J. McGinn*

Aquatic and Crop Resource Development Research Centre, National Research Council Canada, 1411 Oxford Street, Halifax, NS B3H 3Z1, Canada

* Correspondence: arjun.banskota@nrc-cnrc.gc.ca; Tel.: +1-902-426-7372

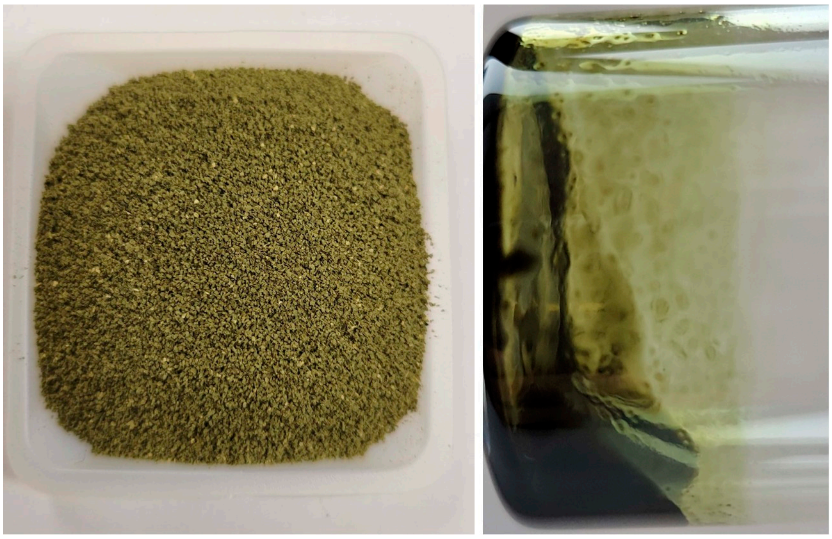

Figure S1. Freeze-dried algal biomass of *M. inermum* (left) and total lipid extract (right).

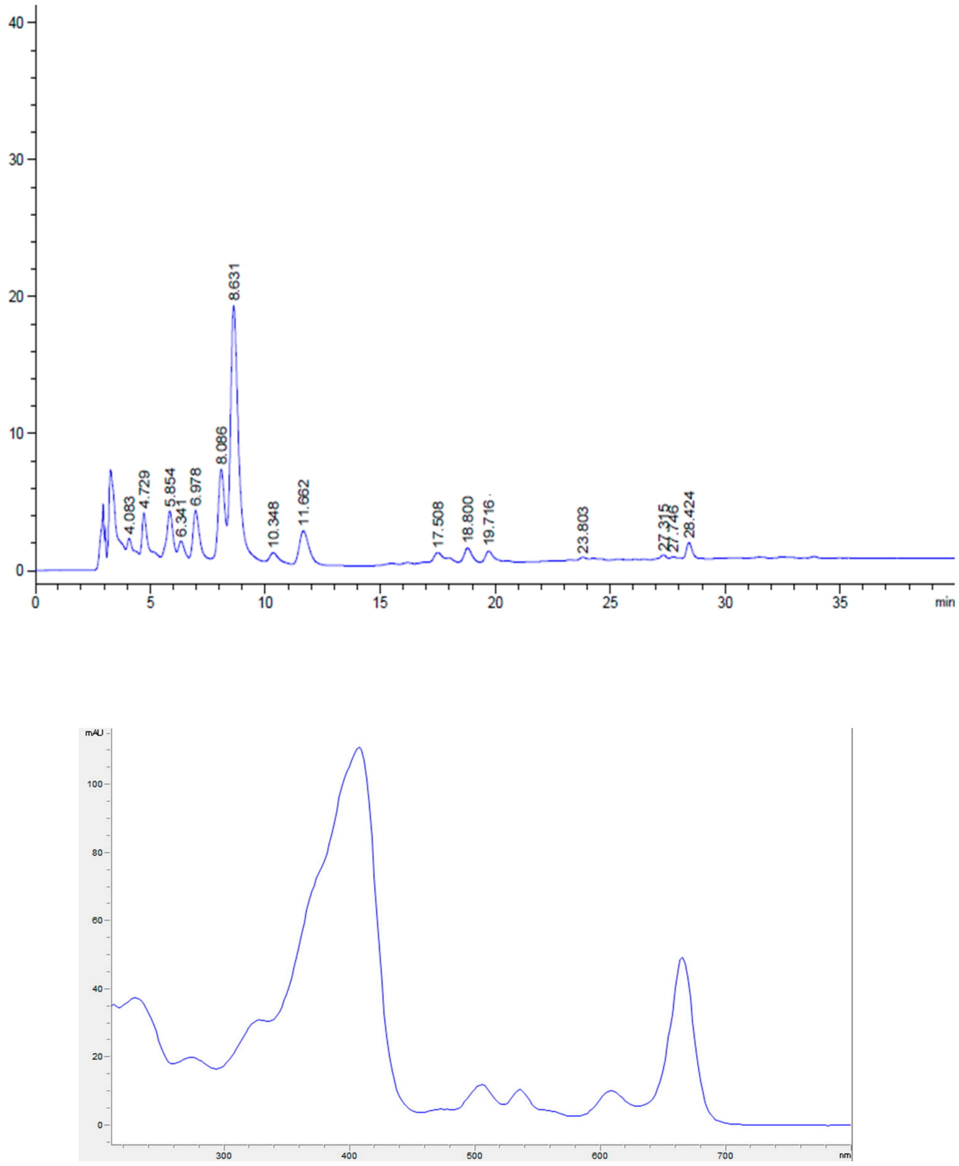

Figure S2. HPLC chromatogram (top) of *M. inermum* lipid at 620 nm and UV spectrum of peak eluted at 8.63 min (bottom). The chlorophyll analysis was performed using an Agilent 1200 series HPLC with a YMC Carotenoid column (5 µm, 2 × 250 mm, 181 YMC Co. Ltd, Tokyo, Japan) eluting with 50 mM NH_4OAc in MeOH/tertiary butyl methyl ether (TBME) linear gradient 5 to 65%B in 30 min at 0.2 mL min^{-1} flow rate for 60 min. Lipid was dissolved in isopropyl alcohol/methanol 1:1 (10 mg/mL) and injection volume was 5 µL.

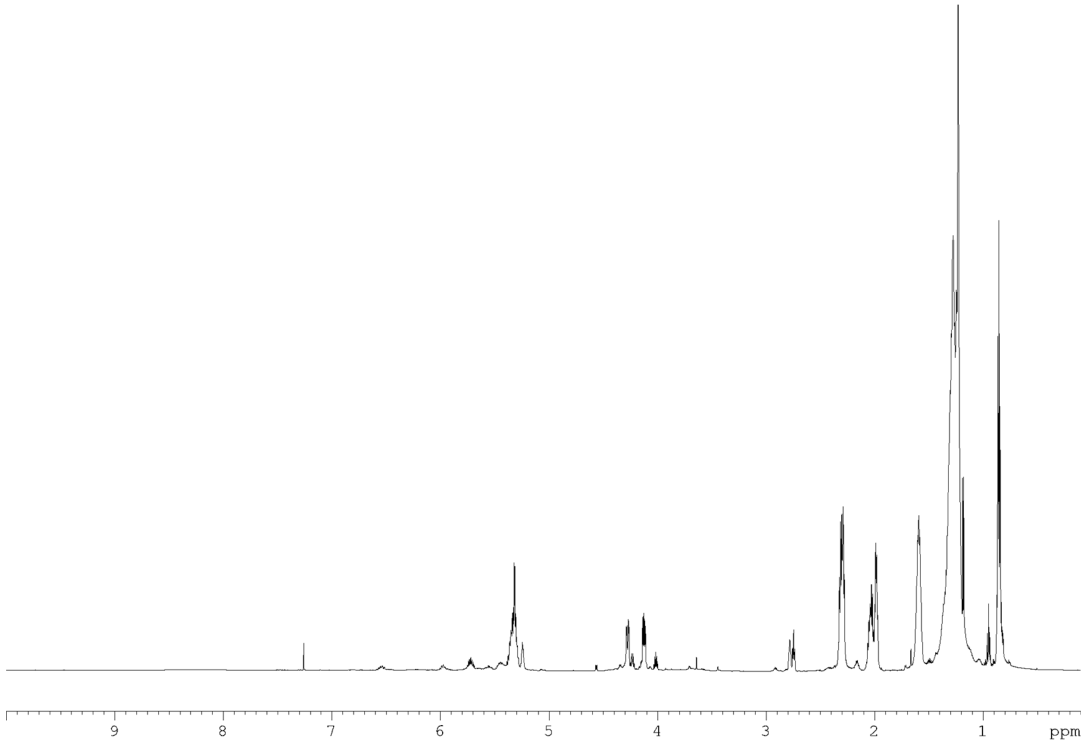

Figure S3. ^{1}H NMR spectrum of total lipid extracted with chloroform/methanol (2:1) recorded in 700 MHz NMR Spectrometer.

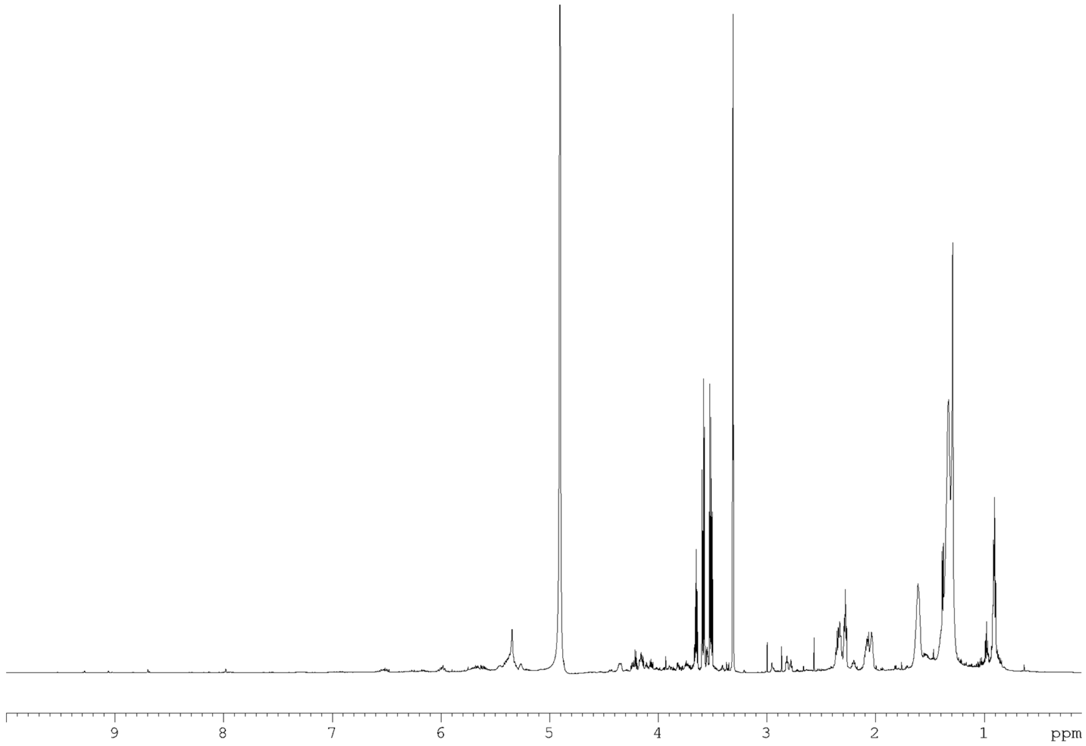

Figure S4. ^{1}H NMR spectrum of glycolipid fraction eluted with acetone in SPE recorded in 700 MHz NMR Spectrometer.

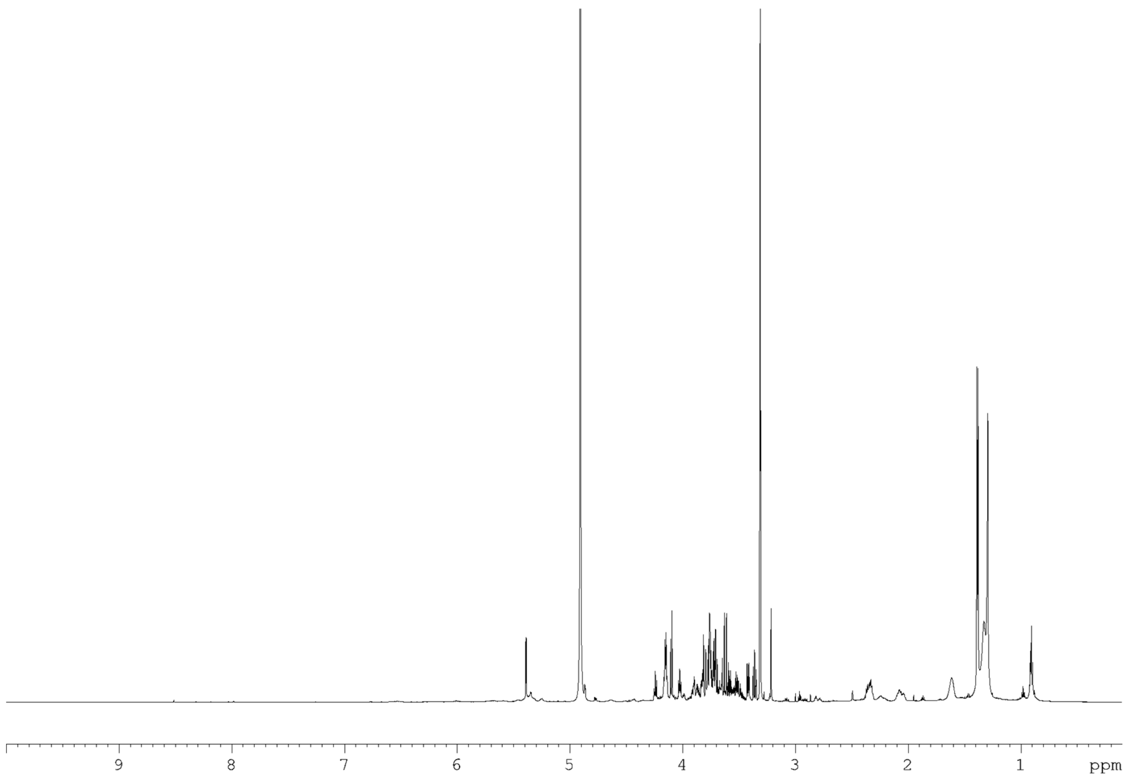

Figure S5. ^{1}H NMR spectrum of phospholipid fraction eluted with methanol in SPE recorded in 700 MHz NMR Spectrometer.

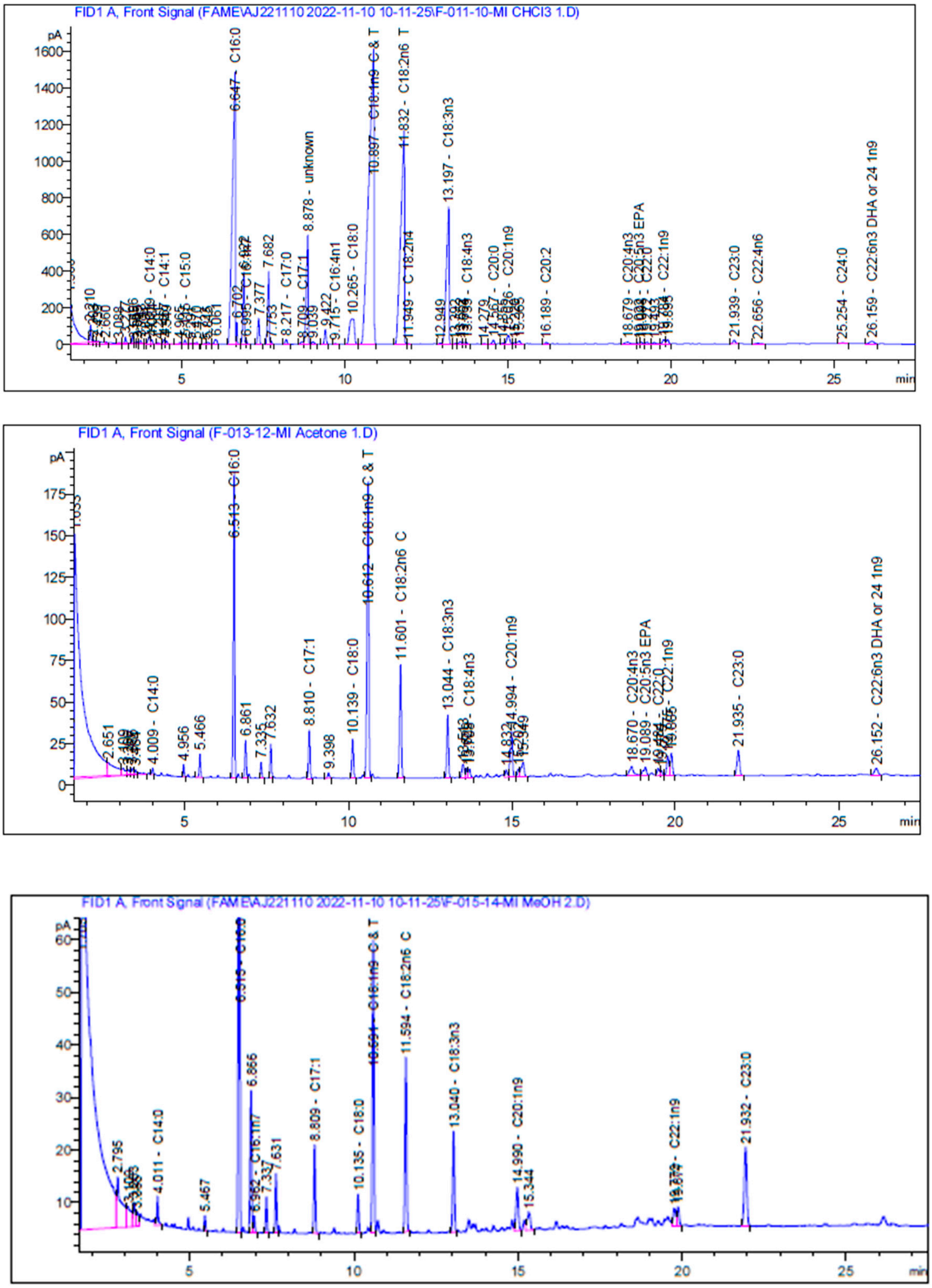

Figure S6. GC chromatogram of neutral lipid (top), glycolipid (middle) and phospholipid (bottom) fractions after transesterification

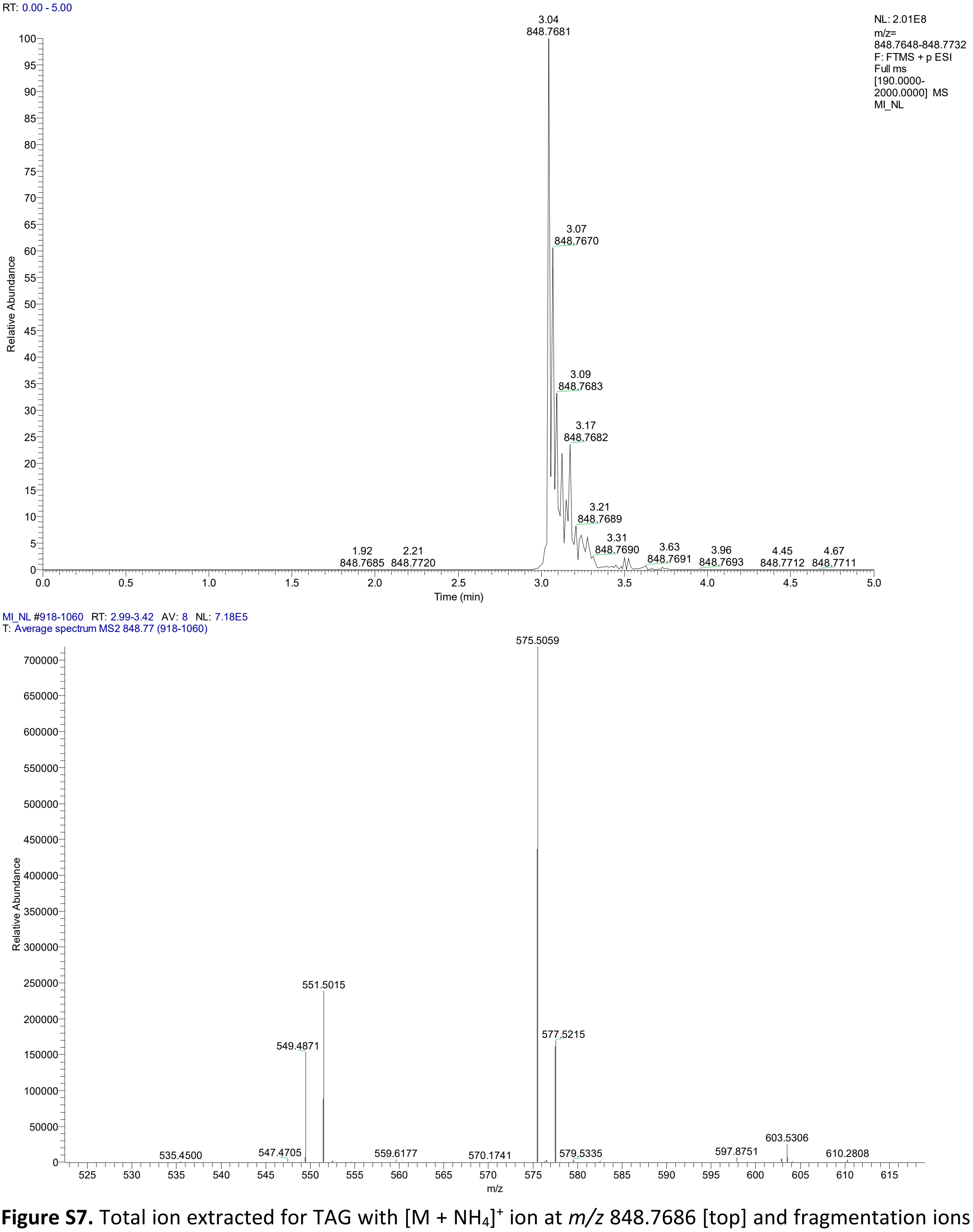

Figure S7. Total ion extracted for TAG with $[M + NH_4]^+$ ion at *m/z* 848.7686 [top] and fragmentation ions corresponding to neutral loss (NL) of TAG (50:2) with their fragmentation ions [bottom]

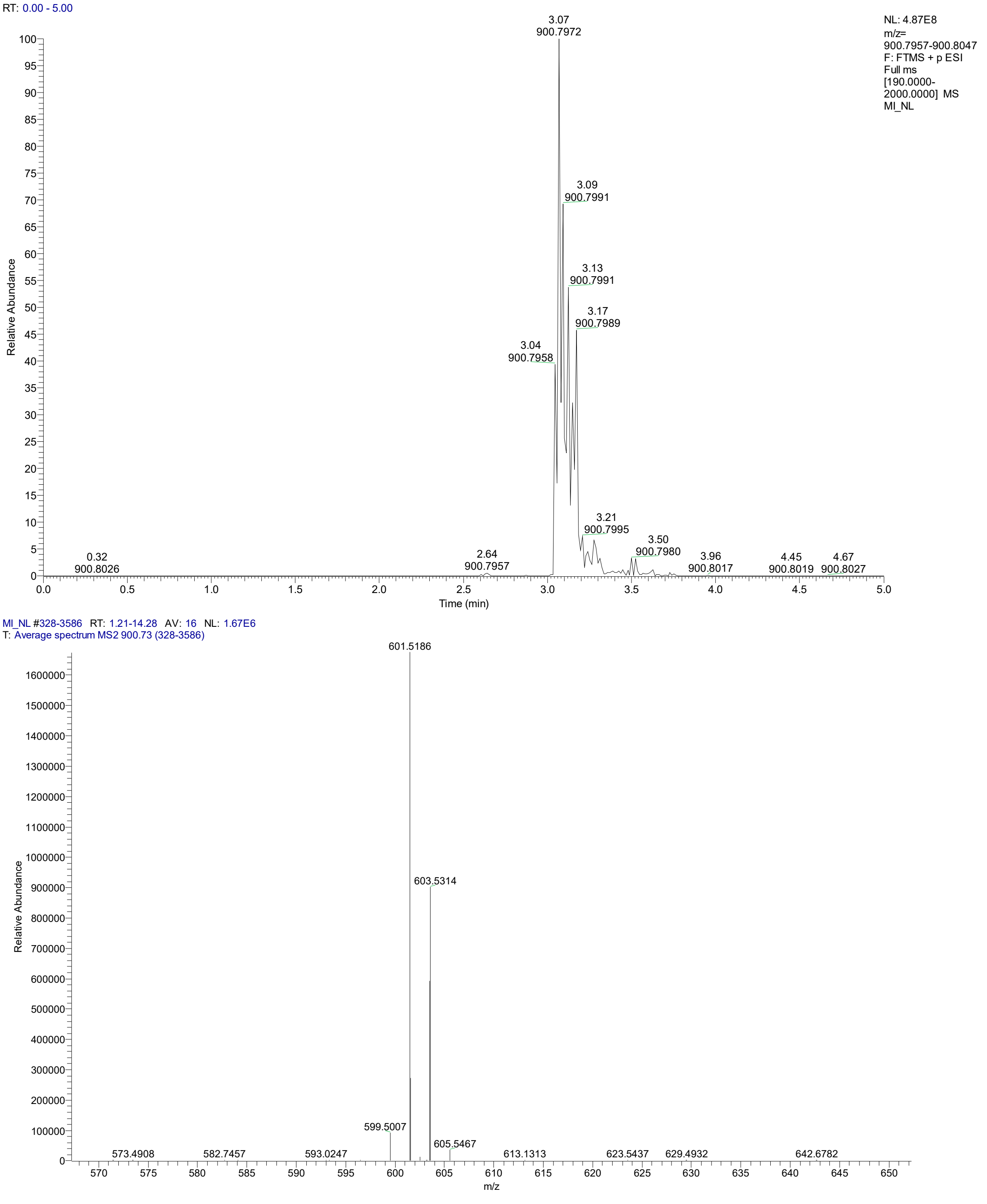

Figure S8. Total ion extracted for TAG with $[M + NH_4]^+$ ion at *m/z* 900.7989 [top] and fragmentation ions corresponding to neutral loss (NL) of TAG (54:4) with their fragmentation ions [bottom]

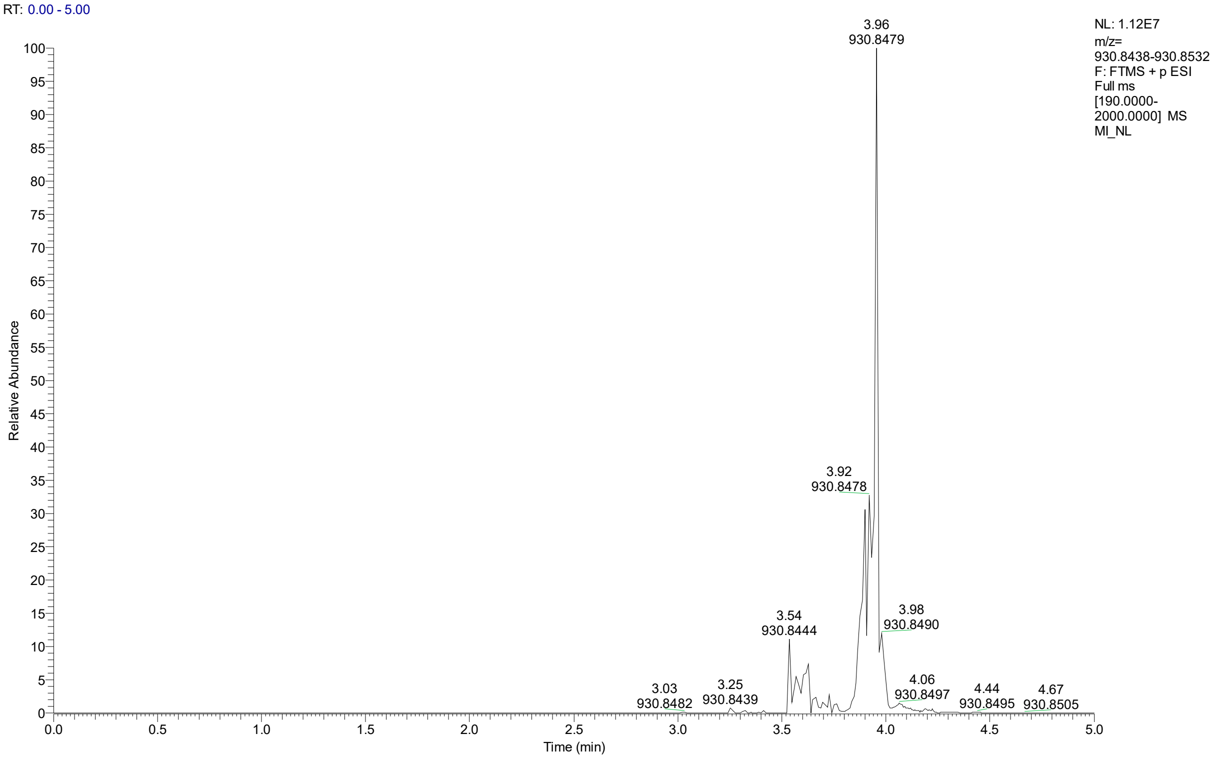

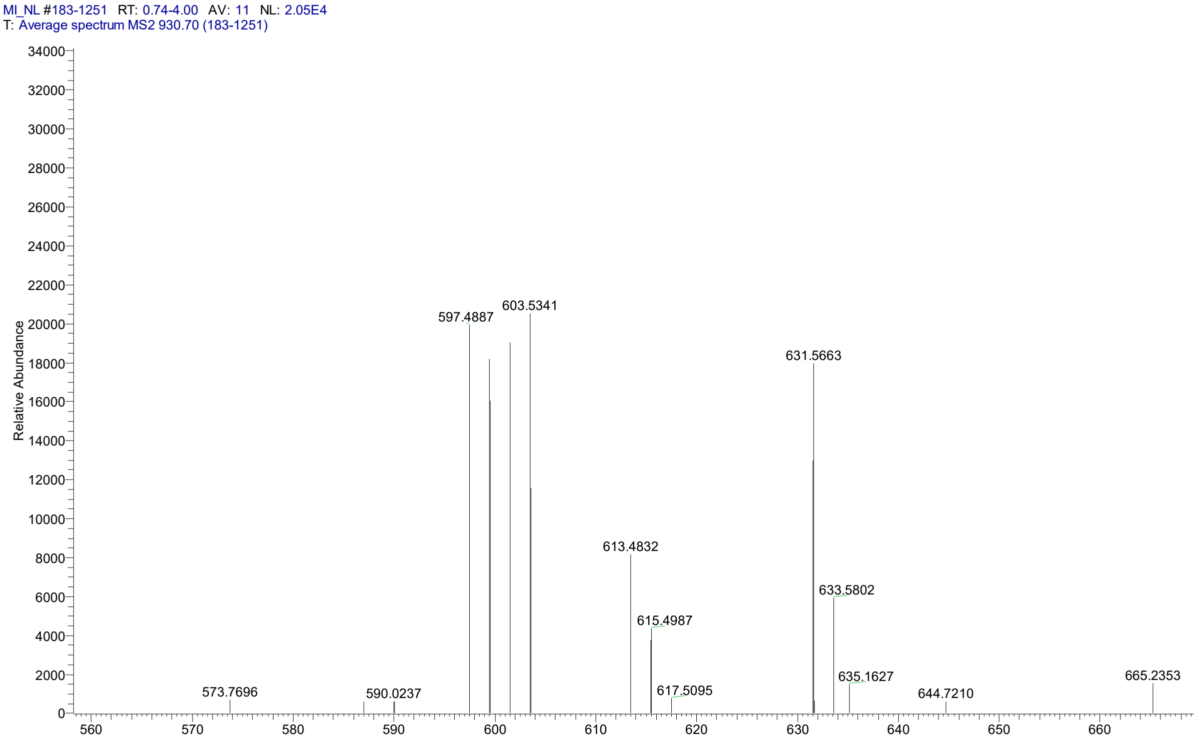

Figure S9. Total ion extracted for unknown TAG with $[M + NH_4]^+$ ion at *m/z* 930.7733 [top] and fragmentation ions corresponding to neutral loss (NL) of TAG (54:5; O2) with their fragmentation ions [bottom]

Table S1. Fatty acid profile of lipid fractions. Results are expressed in mg/g biomass and the percentage of individual fatty acid in the oil is given in the parenthesis.

Fatty acid	*Neutral Lipid (Chloroform Fr)*	*Glycolipid (Acetone Fr)*	*Phopholipid (MeOH Fr)*
Myristic acid	2.4±0.3 (0.5)	1.3±0.1 (0.6)	1.3 (1.5)
Myristoleic acid	1.1± 0.2 (0.2)	-	-
Pentadecanoic acid	0.8±0.0 (0.2)	-	-
cis-10-Pentadecenoic acid	-	-	-
Palmitic acid	103.7±5.0 (21.2)	56.1±0.7 (24.9)	45.4 (49.8)
Palmitoleic acid	0.7±0.0 (0.4)	0.9±0.2 (0.4)	0.8 (0.8)
C16:2 n-4	-	-	-
Heptadecanoic acid	1.2±0.1 (0.2)	-	-
cis-10-Heptadecenoic acid	0.8±0.0 (0.2)	9.3±0.8 (4.1)	4.5 (4.9)
C16:4 n-1	0.2±0.0 (0.0)	-	-
Stearic acid	8.6±0.1 (1.8)	8.8±0.0 (3.9)	2.1 (2.3)
Oleic acid	203.6±14.4 (41.7)	69.7±0.8 (30.9)	16.4 (18.0)
C18:1 n-7	-	1.1±0.2 (0.5)	-
Linoleic acid	105.2±4.5 (21.5)	27.6±1.7 (12.2)	9.9 (10.9)
C18:2 n-4	0.2±0.0 (0.0)	-	-
r-linolenic acid	-	-	-
C18:3 n-4	0.1±0.1 (0.0)	-	-
a-linolenic acid	49.3±0.9 (10.1)	14.8±0.2 (6.6)	5.9 (6.5)
C18:4 n-3	0.4±0.1 (0.1)	2.2±0.2 (1.0)	
C18:4 n-1	-	-	-
Arachidic acid	1.1±0.1 (0.2)	-	-
cis-11-Eicosenoic acid	3.6±0.4 (0.7)	14.3±0.5 (6.3)	3.5 (3.9)
cis-11,14-Eicosadienoic acid	0.5±0.0 (0.1)	-	-
cis-8,11,14-Eicosatrienoic acid	-	-	-
Henicosanoic acid	-	-	-
Arachidonic acid	-	-	-
cis-8,11,14-Eicosatrienoic acid	-	-	-
C20:4 n-3	1.1±0.1 (0.2)	5.2±0.3 (2.3)	-
Eicosapentaenoic acid	0.3±0.0 (0.1)	3.2±1.0 (1.4)	-
Behenic acid	0.6±0.1 (0.1)	2.1±0.2 (0.9)	-
Erucic acid	1.2±0.1 (0.2)	5.8±0.1 (2.6)	1.3 (1.4)
Docosadienoic acid	-	-	-
C22:4 n-6	0.3±0.0 (0.1)	-	-
Lignoceric acid	0.5±0.0 (0.1)	-	-
C24:1 n-9	1.5±0.2 (0.3)	3.1±0.2 (1.4)	-
SFA	**118.9 (24.3)**	**68.3 (30.3)**	**48.9 (53.6)**
MUFA	**212.4 (43.5)**	**103.1 (45.7)**	**26.5 (29.1)**
PUFA	**157.5 (32.2)**	**54.0 (24.0)**	**15.8 (17.3)**
Total FA mg/g (%)	**488.8 (100.0)**	**225.5 (100.0)**	**91.2 (100.0)**

- not detected, Fatty acid analysis was done on duplicate except phospholipid fraction in single experiment.
